# Public health round-up

**DOI:** 10.2471/BLT.18.010318

**Published:** 2018-03-01

**Mobile health clinics: reaching people in need**

A mother taking her child to a mobile health clinic after they were displaced during the conflict in the city of Marawi in the Philippines in 2017. This is one of many mobile clinics deployed in emergencies all over the world by the World Health Organization (WHO) and its partners. bit.ly/2EzcATb

**Figure:**
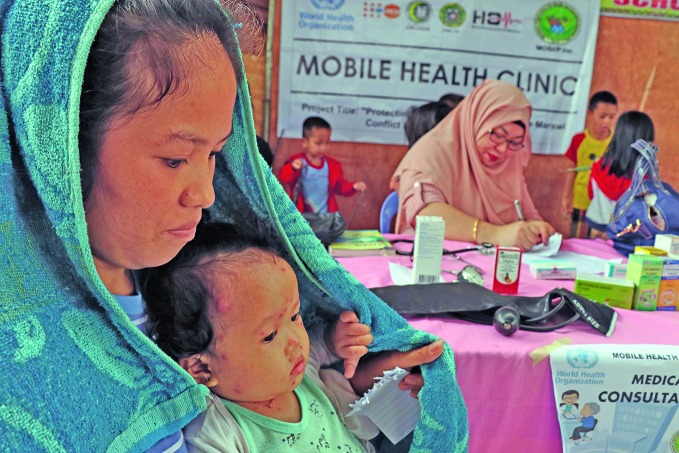
WHO/Faizza Tanggol

## Surveillance of antimicrobial resistance

Bacterial infections in people living in many countries show high levels of antibiotic resistance, according to data released from the World Health Organization’s (WHO) new Global Antimicrobial Surveillance System.

The data – the first to be released since Global Antimicrobial Surveillance System was set up last year – reveal widespread occurrence of antibiotic resistance in a range of bacterial infections in 500 000 people across 22 countries.

The most commonly reported resistant bacteria were Escherichia* coli*, Klebsiella *pneumoniae*, Staphylococcus* aureus*, and Streptococcus *pneumoniae*, followed by Salmonella* spp*.

The surveillance system does not include data on resistance of Mycobacterium *tuberculosis*, because WHO has been tracking this pathogen since 1994 and providing annual updates on drug resistance in the *Global tuberculosis report*.

Among patients with suspected bloodstream infection, the proportion that had bacteria resistant to at least one of the most commonly used antibiotics ranged tremendously between different countries, from zero to 82%.

Resistance to penicillin – the medicine that has been used for decades to treat pneumonia – ranged from zero to 51% among reporting countries. Moreover, between 8% and 65% of E.* coli* isolates associated with urinary tract infections showed resistance to ciprofloxacin, an antibiotic commonly used to treat this condition.

http://bit.ly/2FZlMgS

## Economic losses to cancer

An estimated US$ 46.3 billion was lost in productivity in 2012 due to premature cancer deaths in Brazil, China, India, the Russian Federation and South Africa, known as BRICS countries, according to a study released ahead of World Cancer Day on 4 February.

The BRICS countries account for more than 40% of the world’s population, 25% of the global gross domestic product and 42% of the world’s cancer deaths.

These countries have undergone rapid demographic and economic growth in recent years, and are particularly affected by hepatitis B virus infections and dietary exposure to aflatoxins, both of which can cause liver cancer.

Across the BRICS countries, liver and lung cancer have the largest impact on total productivity lost, the study found. The largest total productivity loss (US$ 28 billion) was in China, due to the country’s large population and high burden of liver cancer.

In the Russian Federation, liver cancer and cancers of the head and neck were probably associated with high consumption of alcohol, the study found.

In India, the use of chewing tobacco was a leading cause of economic loss due to premature mortality from cancers of the lip and oral cavity.

The study, led by the International Agency for Research on Cancer was published in *Cancer Epidemiology*. bit.ly/2o27UuS

## Low influenza vaccine uptake in Europe

Influenza vaccination coverage among older persons has decreased in several countries in the WHO European Region in recent years. In some countries, access to influenza vaccines remains limited.

This analysis of seasonal influenza vaccine coverage in the Region between 2008–09 and 2014–15 was published by the WHO Regional Office for Europe and the European Centre for Disease Prevention and Control (ECDC).

An estimated 44 000 people die every year of respiratory diseases associated with seasonal influenza in the WHO European Region. According to annual surveys funded by ECDC and WHO, 34 000 (over 75%) of these deaths in Europe are of people aged 65 years or above, and vaccine uptake remains low in this group.

Several countries including France and Germany have seen a rapid increase in severe cases of influenza during the 2017–18 influenza season in western Europe. According to EuroMOMO, the European organization for monitoring excess mortality for public health action, some of these countries are reporting excess mortality among the elderly.

Influenza vaccination is generally recommended for people with chronic illnesses, but coverage for this group was below 40% in most countries that reported on this indicator. Almost all countries recommend influenza vaccination for health-care workers, but most of them reported vaccine uptake in this group to be less than 40%.

http://bit.ly/2C4jo6a

## Funds for crisis in Yemen

The United Nations (UN) has released US$ 9.1 million to WHO to provide urgent health assistance to 630 000 vulnerable people in districts around Sana’a and al-Hudayda in Yemen, including 189 000 internally displaced persons and 441 000 people from host communities.

The funds were released from the new UN Central Emergency Response Fund (CERF) grant on 2 February.

“WHO is working with partners to fill critical gaps in the provision of basic health care, to respond to disease outbreaks, to strengthen disease surveillance, to distribute medical supplies and to deliver life-saving services to mothers and their children,” said Dr Nevio Zagaria, the WHO Representative in Yemen.

Only 50% of Yemen’s health facilities are fully functional and some 16.4 million people in the conflict-torn country require assistance to ensure adequate access to health care with 9.3 million of them in acute need, he said.

As lead of the Health Cluster that coordinates the emergency health response in Yemen, WHO is working with 49 partners to deliver health services to 12.3 million people in need.

http://bit.ly/2EqMDoJ

**Cover photo**

A woman washes clothes in a newly constructed water, sanitation and hygiene (WASH) facility that is supported by the United Nations Children’s Fund (UNICEF) in the Bo District of Sierra Leone. Some 175 WASH facilities, like this one, are being constructed across the West African country with funding from the Department for International Development of the United Kingdom of Great Britain and Northern Ireland. The goal is to reduce disease and deaths caused by poor water and hygiene.

**Figure:**
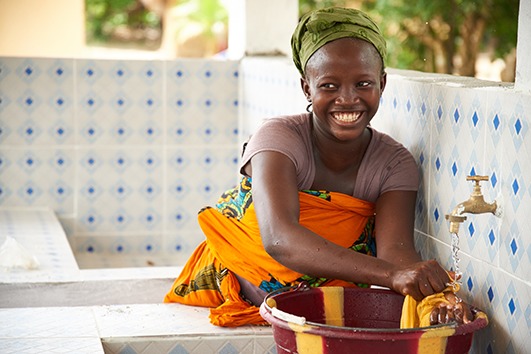
Sam Phelps©UNICEF

## Cholera outbreak ends

South Sudan declared the end of its cholera outbreak on 7 February.

The outbreak was first declared on 22 July 2016 and spread from Juba to 26 other counties of South Sudan, which is facing several complex health emergencies.

The government worked closely with several partners in the outbreak response, including the European Union Humanitarian Aid, the United States Agency for International Development and WHO.

About 2.2 million doses of the oral cholera vaccine were released from the global stockpile that is funded by GAVI, the Vaccine Alliance. Vaccination played a major role in bringing the outbreak under control alongside with water, sanitation and hygiene interventions. 

In 2017, more than 885 000 people were immunized in a first round and nearly 500 000 people also received a second dose of the vaccine.

Due to security challenges, not everyone was able to receive the recommended two doses, which would significantly decrease their risk of being affected by cholera.

By the time the last person with confirmed cholera was discharged on 18 December 2017, more than 20 000 suspected cases and 436 deaths had been reported.

http://www.afro.who.int/news/south-sudan-declares-end-its-longest-cholera-outbreak

## Disparities in health research and development

There are striking disparities in health research investment between countries and topics, according to the Global Observatory on Health Research and Development (R&D).

WHO established the observatory in 2017 to gather data on where and how research funds are being spent so that governments, funders and researchers can use these data to decide how to invest in health research and which topics and countries should take priority.

According to the observatory, the level of investment in certain topics does not correspond to the actual burden of disease.

As little as 1% of all funding for health R&D is allocated to diseases such as malaria and tuberculosis that affect mainly low- and middle-income countries, despite these diseases accounting for more than 12.5% of the global burden of disease.

“Most product-related health R&D is profit-driven. This initiative is important because it shows policy-makers and health research funders that we cannot rely on market forces when it comes to making the investments that are needed in health R&D,” said Taghreed Adam, who works on the observatory.

The 2014 outbreak of Ebola virus disease in West Africa dramatically exposed the lack of investment in prevention and treatment of infections caused by pathogens with epidemic potential, she said.

High-income countries have on average 40 times more health researchers than low-income countries. The number of health research workers per million inhabitants ranges from 1140 in Singapore to 0.2 in Zimbabwe. In high-income countries, an average of one in two health researchers are women, but in low-income countries the average is one in four.

 http://bit.ly/2H7bXPi

**Looking ahead**

7 April – World Health Day devoted to universal health coverage

24–30 April – World Immunization Week

21–26 May – Seventy-first World Health Assembly

31 May – World No Tobacco Day

